# Sensitivity of HOXB13 as a Diagnostic Immunohistochemical Marker of Prostatic Origin in Prostate Cancer Metastases: Comparison to PSA, Prostein, Androgen Receptor, *ERG*, *NKX3.1*, PSAP, and PSMA

**DOI:** 10.3390/ijms18061151

**Published:** 2017-05-29

**Authors:** Ilka Kristiansen, Carsten Stephan, Klaus Jung, Manfred Dietel, Anja Rieger, Yuri Tolkach, Glen Kristiansen

**Affiliations:** 1 Institute of Pathology, University Hospital Bonn, 53127 Bonn, Germany; ilka.kristiansen@gmx.de (I.K.); Yuri.tolkach@ukbonn.de (Y.T.); 2 Department of Urology, Charité-Universitätsmedizin Berlin, 10117 Berlin, Germany; carsten.stephan@charite.de; 3 Berlin Institute of Urologic Research, 10117 Berlin, Germany; klaus.jung@charite.de; 4 Institute of Pathology, Charité-Universitätsmedizin Berlin, 10117 Berlin, Germany; Manfred.dietel@charite.de (M.D.); Anja.rieger@charite.de (A.R.)

**Keywords:** prostate cancer, metastasis, immunohistochemistry, detection, PSA, PSAP, PSMA, NKX3.1, prostein, HOXB13, ERG, AR

## Abstract

Aims: Determining the origin of metastases is an important task of pathologists to allow for the initiation of a tumor-specific therapy. Recently, homeobox protein Hox-B13 (HOXB13) has been suggested as a new marker for the detection of prostatic origin. The aim of this study was to evaluate the diagnostic sensitivity of HOXB13 in comparison to commonly used immunohistochemical markers for prostate cancer. Materials and methods: Histologically confirmed prostate cancer lymph node metastases from 64 cases were used to test the diagnostic value of immunohistochemical markers: prostate specific antigen (PSA), Prostatic acid phosphatase (PSAP), prostate specific membrane antigen (PSMA), homeobox gene *NKX3.1*, prostein, androgen receptor (AR), HOXB13, and ETS-related gene (ERG). All markers were evaluated semi-quantitatively using Remmele’s immune reactive score. Results: The detection rate of prostate origin of metastasis for single markers was 100% for NKX3.1, 98.1% for AR, 84.3% for PSMA, 80.8% for PSA, 66% for PSAP, 60.4% for HOXB13, 59.6% for prostein, and 50.0% for ERG. Conclusions: Our data suggest that HOXB13 on its own lacks sensitivity for the detection of prostatic origin. Therefore, this marker should be only used in conjunction with other markers, preferably the highly specific PSA. The combination of PSA with NKX3.1 shows a higher sensitivity and thus appears preferable in this setting.

## 1. Introduction

Determining the origin of the primary tumor in metastases is an important task of pathologists to allow for the initiation of a tumor-specific therapy. Since its description in the 1970s, prostate specific antigen (PSA) has in due course become the dominant prostate marker in serum [1,2]. PSA could be detected by immunohistochemistry in tissue and was quickly adopted by pathologists as a diagnostic marker [3,4]. PSA is still regarded as a highly specific marker of prostatic origin, but due to its decreased or even lost expression in higher grade or metastatic tumors, which was already noted in initial studies, its sensitivity is clearly limited, which necessitates the use of additional markers in PSA negative cases [5]. Prostein, coded by the *SLC45A3* gene and identified by transcript profiling studies of prostate cancer, was found to be highly specific for prostatic origin and displays a characteristic Golgi-type cytoplasmic staining pattern [6,7]. As seen with PSA, prostein expression also shows a slightly diminished expression with tumor progression, but in combination with PSA it proved to be a valuable tool to identify prostatic origin [8,9]. An alternative marker for prostatic origin was the protein coded by the homeobox gene *NKX3.1*, which shows nuclear staining on immunohistochemistry and which was also found to be highly though not exclusively specific for prostate tissues [10,11]. At the 2014 consensus conference of the International Society of Urological Pathology (ISUP), it was recommended to use immunohistochemistry for PSA, NKX3.1 and prostein to ascertain a prostatic origin in doubtful cases [5].

Another diagnostic candidate marker that was not considered at that conference was homeobox protein Hox-B13 (HOXB13), which was characterized as a marker for prostate cancer by Edwards et al. [12]. HOXB13 also has a nuclear staining pattern and has been recommended by several studies as a prostate specific marker [13,14,15,16]. To date, the diagnostic value and especially the performance of HOXB13 immunohistochemistry as a marker of prostatic origin in direct comparison to the commonly used prostate markers (PSA, prostein, ERG, the androgen receptor (AR), NKX3.1, Prostatic acid phosphatase (PSAP) and prostate specific membrane antigen (PSMA)) in metastases has not been evaluated, which was therefore the aim of this study.

## 2. Results

### 2.1. Immunohistochemical Staining Patterns

**Figure 1:**
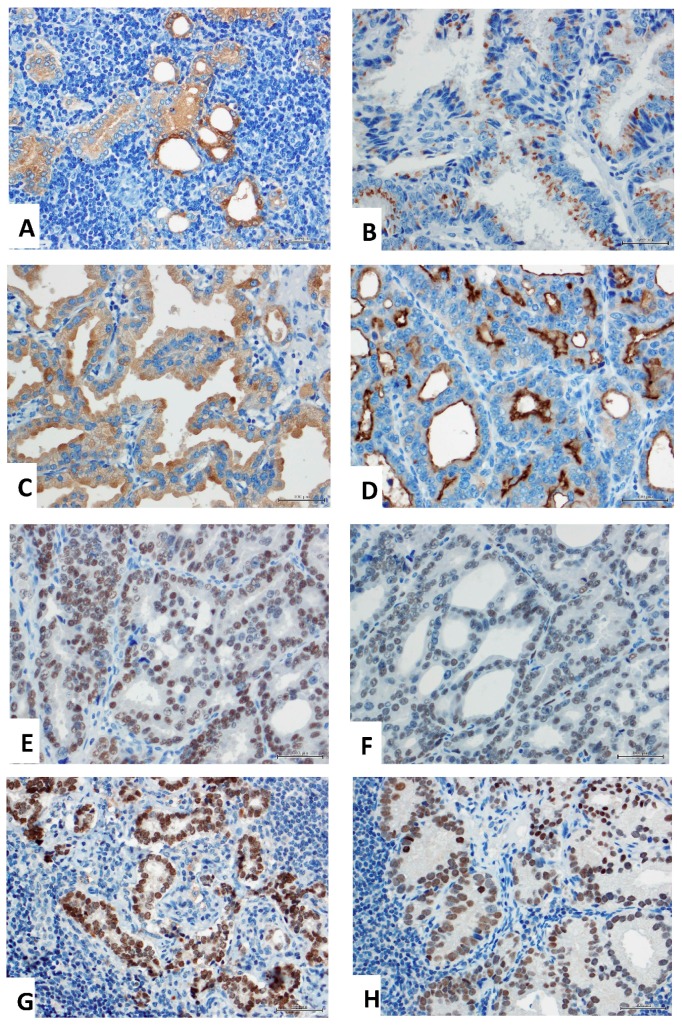
Immunohistochemistry of candidate diagnostic prostate markers. (**A**) Prostate specific antigen (PSA) shows diffuse cytoplasmic staining with a higher degree of heterogeneity in this case. (**B**) Prostein immunoreactivity is restricted to the Golgi apparatus area. (**C**) Prostate specific alkaline phosphatase (PSAP) also displays a diffuse cytoplasmic staining pattern. (**D**) Prostate specific membrane antigen (PSMA) has characteristic cytoplasmic but also membranous staining. (**E**) Androgen receptor shows epithelial nuclear staining. (**F**) ERG is also located in the nucleus and has a higher degree of heterogeneity, as seen here. (**G**) Homeobox gene *NKX3.1* commonly displays strong nuclear staining. (**H**) Homeobox protein HOXB3 is also seen in nuclei, however, the staining intensity is weaker. All images are taken in 400×.

All markers showed the expected patterns of immunoreactivity in metastatic prostate cancer. Prostate specific antigen (PSA) and prostatic phosphatase (PSAP) displayed diffuse cytoplasmic staining (Figure 1A,C), where prostein showed the characteristic Golgi-type staining (Figure 1B). Prostate specific membrane antigen (PSMA, Figure 1D) showed a predominantly membranous but also cytoplasmic immunoreactivity. Nuclear staining was seen for the androgen receptor (AR), ERG, NKX3.1, and HOXB13 (Figure 1E–H).

### 2.2. Statistical Evaluation of Prostate Markers

The highest detection rate was seen with NKX3.1 followed by the androgen receptor and PSMA. PSA, as the most commonly used antibody to specifically recognize prostate cancer, correctly detected nearly 81% of cases. ERG labeled, as expected, only 50% of prostate cancer metastases. Prostein, PSAP, and HOXB13 were only slightly superior, detecting approximately two thirds of cases (Table 1).

A Spearman rank correlation analysis of IRS values of these markers revealed the following significant associations: AR and HOXB13 (correlation coefficient (CC) = 0.358, *p* = 0.009), AR and NKX3.1 (CC = 0.505, *p* = 0.001), AR and PSAP (CC = −0.277, *p* = 0.047), ERG and HOXB13 (CC = 0.283, *p* = 0.044), PSA and Prostein (CC = 0.367, *p* = 0.008), PSA and PSAP (CC = 0.623, *p* = 0.001), PSA and PSMA (CC = 0.350, *p* = 0.012), prostein and PSAP (CC = 0.277, *p* = 0.049), and prostein and HOXB13 (CC = −0.296, *p* = 0.035).

We then investigated which combination of prostate markers would achieve the highest detection rate by combining the most commonly used marker PSA with the other markers in cross tables. PSAP recognized 10% of PSA negative cases, which did not add significant information. PSMA performed slightly better, labelling 50% of PSA negative cases. HOXB13 correctly recognized 70%, and finally *NKX3.1* correctly detected all PSA negative cases, leading to a detection rate (of the combination of PSA and *NKX3.1*) of 100% in this dataset. Prostein and PSA correctly detected 82% of cases, HOXB13 and PSA labeled 94.2% of cases correctly, AR and PSA detected 100% of cases, and ERG and PSA recognized 88% of prostate cancer metastases.

In a direct analysis of HOXB13 and NKX3.1 expression, both markers together recognized 100%, as NKX3.1 labeled 100% of HOXB13 negative cases. NKX3.1 combined with AR also achieved a detection rate of 100%.

## 3. Material and Methods

### 3.1. Case Selection and Construction of Tissue Microarray

First, prostate cancer patients who underwent radical prostatectomy (RP) and lymphadenectomy and who were diagnosed at the Institute of Pathology, Charité–Universitätsmedizin Berlin between 1998 and 2005 were identified. Histologically confirmed metastases from 64 cases were used for tissue microarray construction with one spot per case and a punch size of 1 mm. The tissue microarray comprised of two blocks. The metastases consisted of 57 local lymph node metastases and seven systemic metastases (4× bone, 1× penis, 1× oral mucosa, 1× cervical lymph node). Lymph nodes were submitted to frozen section analysis prior to RP, which was usually not performed in case of a positive node. Only 13 RPs were performed. Therefore, histopathological RP data on the primary tumor was not available for the majority of cases. For the thirteen RP cases that were completed, the Gleason scores (GS) were: GS7-2, GS8-4, GS9-4, GS10-3. The pT categories (according to Union for International Cancer Control (UICC) TNM classification of malignant tumors, 8th edition) were: pT2-2, pT3a-1, pT3b-10. Positive margins were seen in 11 cases. The bilateral lymphonodectomy specimens (*n* = 57) contained a median of 12 lymph nodes (range 1 to 23). Twenty-nine cases had a single positive lymph node, 15 cases had two metastases, seven cases had three metastases and six cases had four or more positive nodes (maximum 11). The use of this tissue was approved by the Charité University Ethics Committee (EA1/06/2004). No animals were involved in this study.

### 3.2. Immunohistochemistry

Immunohistochemistry was conducted using semi-automated platforms (Benchmark Ultra, Roche, and Autostainer, Medac) with protocols that are in routine use at the Institute of Pathology, University of Bonn (Table 2).

### 3.3. Evaluation of Immunohistochemistry

All markers were evaluated semi-quantitatively using the immune reactive score (IRS), which gives the product of categorized percentage of stained cells (0: negative, 1: 1–10%, 2: 11–50%, 3: 51–80%, and 4: >80%) and staining intensity (ranging from negative (0) to strong (3)), and hence has a range from 0 to 12. For statistical evaluation, an IRS below 3 was considered a negative test and higher values were considered as positive, as suggested by Remmele and Stegner in their original proposal of the IRS scoring system [17]. The evaluation was carried out under the immediate supervision of a Genito–Urinary pathologist with broad expertise in immunohistochemistry (Glen Kristiansen).

### 3.4. Statistics

All data were processed using SPSS (IBM SPSS Statistics for Macintosh, Version 22.0, IBM Corp., Armonk, NY, USA). Spearman Rank correlations were used to analyze the associations among markers.

## 4. Discussion

This study evaluates the sensitivity of prostate markers to detect lymph node and distant metastases of prostate cancer. It confirms that PSA, as the most commonly used antibody for routine purposes, has a relatively high detection rate of nearly 81%. The value of PSA is its high specificity, as apart from prostate cancer only breast cancer may be PSA positive, but this is usually not in the closer differential diagnosis [18]. The loss of PSA expression with tumor dedifferentiation and progression is long known and constitutes the necessity to use other markers in a combination.

As prostate cancer is an androgen-driven disease, the high rates of androgen receptor expression found in primary and metastatic prostate tumors are not surprising, and again this study confirms that metastases retain their AR expression well. The disadvantage of AR is the relative lack of specificity, as other neoplasms including urothelial carcinoma or salivary gland tumors may also be AR positive [19,20].

The overexpression of ERG, often resulting from the Transmembrane protease, serine 2 - erythroblast transformation-specific-related gene TMPRSS2-ERG translocation which is found in nearly half of primary prostate cancer cases, is therefore typical of prostate cancer, but with a detection rate of 50% it clearly lacks sensitivity [21]. It is less well known that even though the genomic translocation is highly specific for prostate cancer, ERG protein expression is seen in a variety of other tumors, too. This includes, apart from vascular tumors, round cell sarcomas and leukemias, the more common differential diagnosis of urothelial carcinoma and, according to the human protein atlas (hppt://www.proteinatlas.org), also melanoma, testicular, and gastrointestinal tumors [22,23,24].

Prostein was described and characterized by Xu et al. as a prostate specific protein, which was quickly used by surgical pathologists for the differential diagnosis of prostate cancer, especially to rule out urothelial carcinoma, which is almost consistently prostein-negative [6,7,9,25,26]. Prostein also has the advantage of a distinctive Golgi-type staining pattern, which can be reassuring in cases with only weak positivity. In primary prostate cancer, prostein expression is inversely correlated with Gleason scores and is a prognostic marker of disease progression [27]. As our study confirms, the sensitivity of prostein in metastases is fairly limited with a detection rate of 59%, and even in conjunction with PSA only 82% of cases can be confirmed as prostatic in origin, which equals the detection rate of PSA alone in our cohort. As we found that both markers correlated, this redundancy of diagnostic information is not surprising. The same holds true for PSAP, which we found strongly and highly positively correlated to PSA, and this did not add significant information as only 10% of PSA negative cases were picked up by PSAP. A larger cohort may be necessary to demonstrate the additional diagnostic value of prostein or PSAP to PSA.

Prostate specific membrane antigen (PSMA) is overexpressed during tumor progression, and Bostwick et al. found 82% positivity rates in primary prostate cancer [28]. However, despite its name it is not prostate specific at all, but is also seen in a wide variety of tumors including colon, bladder, and renal cancer, so its use as a marker for prostatic differentiation is now discouraged [29].

NKX3.1 is a homeobox gene that shows a prostate and testis specific expression pattern, but may also be found in breast cancer. Its crisp nuclear staining pattern and its high rate of positivity in metastases (98.6%, Gurel et al. [30]), which this study confirms, have made it a popular diagnostic marker that is already endorsed by ISUP in their recommendations on diagnostic markers for genito–urinary pathology [11,30,31,32,33]. In particular, its combination with PSA is promising, as the high detection rate of 100% found in this study witnesses.

HOXB13 is another, so far less acknowledged diagnostic marker candidate, which has been recommended lately as a prostate specific marker [12,13,15,16]. Barresi et al. analyzed 15 prostate cancer metastases and found all of them strongly positive (in >75% of tumor cells) which equates a sensitivity of 100% [15]. Minimally lower rates were reported by Varinot et al., who analyzed 74 cases of lymph node metastases and 15 additional bone metastases. They found HOXB13 positivity in 33% of bone metastases and 93% of lymph node metastases. Interestingly, the expression of HOXB13 was also found as an independent prognostic marker in primary prostate cancer and to correlate with AR expression [14]. While our study confirms the significant association with AR, the diagnostic value of HOBX13 appears less convincing, as only 60.4% of prostate cancer metastases were positively stained. Of course, besides aspects of cohort composition that may influence the tumor biology, technical issues of the immunohistochemistry protocol or the tissue micro array construction may also explain this rather significant discrepancy. This detection rate increased markedly to 94.2% if combined with PSA, but it remains inferior to the combination of PSA with NKX3.1. HOXB13 is also expressed in other carcinomas including endometrial cancer, which in itself does not limit its diagnostic value, but also in pancreatic cancer and hepatocellular carcinoma [34,35,36]. In light of these data, we do not recommend HOXB13 as a sole marker to detect a prostatic origin in a metastasis, but rather prefer PSA in conjunction with NKX3.1. However, prostein or HOXB13 may well be considered as third line markers in doubtful cases, and here combinations are advisable and often necessary [5,37].

This study has several weaknesses. The number of cases is relatively small, which precludes a more detailed analysis of combinational subgroups. Still, the cohort size is large enough to confirm the known expression rates of the markers under question and it is the first study to critically analyze HOXB13 in direct comparison to these other markers in prostate cancer metastases. This study is restricted to the correct detection of prostate markers in known prostate metastases and hence only evaluates the sensitivity of these markers. Also, this study lacks data on the Gleason scores of biopsies of primary tumors, which precludes further correlation analyses of biomarker expression. Finally, we did not aim to verify the specificity of our candidate biomarkers, which would have necessitated an additional large analysis of non-prostatic neoplasms [38].

In summary, our data suggest that the novel marker candidate HOXB13 alone is not a good diagnostic marker for the detection of prostatic origin. Only in combination with PSA does it achieve satisfactory detection rates. Alternatively, the combination of PSA with the already well-established marker NKX3.1 shows an even higher sensitivity and is therefore recommended in this setting.

## Figures and Tables

**Table 1:** Detection rates of prostate markers in prostate cancer metastases.

Marker	Detection Rate (%)	Mean IRS	Number of Cases
PSA	80.8	6.3	52
PSAP	66.0	3.5	53
PSMA	84.3	6.0	51
Prostein	59.6	4.2	52
Androgen receptor (AR)	98.1	6.7	53
*ERG*	50.0	2.6	52
*NKX3.1*	100.0	8.0	50
HOXB13	60.4	4.7	53

Abbreviations: IRS = immunoreactive Score according to Remmele.

**Table 2:** Antibodies used for immunohistochemistry in this study.

Antigen	Clone	Provider	Dilution	Platform	Protocol
PSA	Polyclonal, rabbit	DAKO	1:20,000	Autostainer	No pretreatment
PSAP	PASE/4LJ	Cell Marque	1:6000	Autostainer	HIER (pH 6, 20 s 98 ℃)
PSMA	3E6	DAKO	1:500	Benchmark	CC1 (pH 8), ultraview
Prostein	10E3	DAKO	1:100	Benchmark	CC1 (pH 8), ultraview
Androgen Receptor (AR)	AR441	DAKO	1:400	Autostainer	HIER (pH 6, 20 s 98 ℃)
ERG	EPR3864	Biologo	1:100	Autostainer	HIER (pH 6, 20 s 98 ℃)
NKX3.1	EP356	Cell Marque	1:200	Benchmark	CC1 (pH 8), ultraview
HOXB13	F-9	Santa Cruz	1:50	Benchmark	CC1 (pH 8), optiview

Abbreviations: HIER-Heat Induced Epitope Retrieval.
